# Cerebral Vasomotor Reactivity in COVID-19: A Narrative Review

**DOI:** 10.3390/life13071614

**Published:** 2023-07-24

**Authors:** Zahra Ghotbi, Mehrdad Estakhr, Melika Hosseini, Reza Bavarsad Shahripour

**Affiliations:** 1 Clinical Neurology Research Center, Shiraz University of Medical Sciences, Shiraz P.O. Box 71348-14336, Iran; neuro.ghotbi@gmail.com (Z.G.); mehrdadestakhr@gmail.com (M.E.); 2 Comprehensive Stroke Center, Department of Neurosciences, Loma Linda University, Loma Linda, CA 92354, USA; mhosseini@llu.edu; 3 UCSD Comprehensive Stroke Center, Department of Neurosciences, University of California, San Diego, CA 92093, USA

**Keywords:** SARS-CoV-2, post-COVID complications, vasomotor reactivity, transcranial color doppler, neuromonitoring, VMR

## Abstract

Severe acute respiratory syndrome coronavirus 2 (SARS-CoV-2) primarily affects the respiratory system but can also lead to neurological complications. Among COVID-19 patients, the endothelium is considered the Achilles heel. A variety of endothelial dysfunctions may result from SARS-CoV-2 infection and subsequent endotheliitis, such as altered vascular tone, oxidative stress, and cytokine storms. The cerebral hemodynamic impairment that is caused is associated with a higher probability of severe disease and poor outcomes in patients with COVID-19. This review summarizes the most relevant literature on the role of vasomotor reactivity (VMR) in COVID-19 patients. An overview of the research articles is presented. Most of the studies have supported the hypothesis that endothelial dysfunction and cerebral VMR impairment occur in COVID-19 patients. Researchers believe these alterations may be due to direct viral invasion of the brain or indirect effects, such as inflammation and cytokines. Recently, researchers have concluded that viruses such as the Human Herpes Virus 8 and the Hantavirus predominantly affect endothelial cells and, therefore, affect cerebral hemodynamics. Especially in COVID-19 patients, impaired VMR is associated with a higher risk of severe disease and poor outcomes. Using VMR, one can gain valuable insight into a patient’s disease progression and make more informed decisions regarding appropriate treatment options. A new pandemic may develop with the COVID-19 virus or other viruses, making it essential that healthcare providers and researchers remain focused on developing new strategies for improving survival in such patients, particularly those with cerebrovascular risk factors.

## 1. Introduction

COVID-19 has become one of the leading causes of death worldwide, making it one of the most devastating health issues of the past few decades [1]. Despite the primary target of COVID-19 being the respiratory system, mounting evidence indicates that it may also negatively impact the cerebrovascular system [2,3]. In addition to respiratory symptoms, reports of neurological manifestations of severe acute respiratory syndrome coronavirus 2 (SARS-CoV-2) are emerging. These neurological manifestations include headache, ageusia (loss of taste), anosmia (loss of smell), as well as severe complications including seizures, ischemic stroke, cerebral hemorrhage, encephalitis, and meningitis [4,5,6]. Patients who suffer from severe clinical manifestations of SARS-CoV-2 infection are more likely to experience neurological symptoms compared to those who suffer from mild symptoms [7]. This virus is distinguished by the presence of the spike (S) glycoprotein, which provides the virus with access to neural, glial, and endothelial cells containing angiotensin-converting enzyme 2 (ACE2) [8]. There is still much to be discovered about the pathophysiology of this virus and how it affects the nervous system. However, it is believed that the neurological manifestations of acute COVID-19 can be attributed to multiple overlapping pathogenetic mechanisms [9]. These include viral neuroinvasion, endotheliopathy associated with blood–brain barrier dysfunction, coagulopathies that precipitate hypoxic-ischemic neuronal damage, metabolic imbalances, oxidative stress cascades, and cellular apoptosis [10].

In COVID-19 patients, the endothelial cells are regarded as the Achilles heel since injury to the endothelium will initiate and propagate SARS-CoV-2 infection [11]. The endothelium produces substances that cause blood vessels to contract or relax, which corresponds to cerebral hemodynamic vasomotor reactivity (VMR) [12]. With vasodilator stimulation, such as CO_2_ inhalation, breath-holding test (BHT), or acetazolamide administration, VMR can be assessed using rates of blood flow in cerebral arteries and changes in blood flow caused by hypercarbia [13]. The impairment of vasoreactivity and reduced reserve capacity in brain arteries predispose patients to cerebrovascular disease. Recently, researchers have found that impaired VMR is associated with a higher probability of severe illness and poor outcomes in patients with COVID-19 [14]. It is worth mentioning that structural changes in vasculature occur more slowly than functional changes. Hence, functional assessment of the vasculature, such as the VMR assessment, is more sensitive than structural analysis when acute exposure to the disease process is present [15]. Researchers have shown that viruses other than SARS-CoV-2, such as the Human Herpesvirus 8 and Hantavirus, can negatively impact cerebral hemodynamics [16]. In treating patients with viral infections such as COVID-19, physicians should consider VMR because of its potential to implement an appropriate, speedy, and aggressive treatment to improve neurological sequelae. Additionally, VMR has been observed to change over time in patients with different clinical neurologic manifestations, suggesting it could be a biomarker for the disease’s progression [14]. It may be possible to prescribe targeted interventions to patients who might benefit from them, resulting in improved patient outcomes. Furthermore, it would reduce the burden associated with cerebrovascular disorders by utilizing valuable information obtained from a VMR evaluation.

This review summarizes the most relevant literature on cerebral hemodynamic changes, particularly VMR impairment in COVID-19 patients in the acute or chronic infection phases.

## 2. COVID-19 Infection and Cerebral Vascular Health

There are a variety of neurological manifestations associated with COVID-19, ranging from minor symptoms like dizziness, headache, and loss or disruption of the sense of smell (anosmia/dysosmia) and taste (ageusia/dysgeusia), as well as severe conditions like stroke, Guillain-Barré syndrome (GBS), acute hemorrhagic necrotizing encephalopathy, and cerebral venous thrombosis [10]. ACE2 receptors enable SARS-CoV-2 to enter host cells via its spike protein [6]. It is reported that these receptors are highly expressed in various tissues, such as the heart, lungs, respiratory tract epithelium, endothelial cells, and brain [17]. The fusion of viral and cellular membranes is initiated by the spike protein, which is activated by the serine protease TMPRSS2. This fusion process leads to virus and receptor internalization, representing the initial step of cellular infection [18]. The spike protein in SARS-CoV-2 exhibits a higher binding affinity to ACE2 than SARS-CoV. Consequently, this increased affinity enhances the potential of SARS-CoV-2 to infect brain cells expressing ACE2 [19]. 

Evidence suggests that coronavirus can penetrate the brain, infecting neurons and glial cells [20,21]. The presence of central nervous system (CNS) coronavirus infection has been detected in the neural cells and cerebrospinal fluid (CSF) of individuals affected by COVID-19 [22]. ACE2 is expressed in distinct neuronal groups within the brain and brainstem [23]. Given that neurons possess a unique chemical signature, any alteration in this signature may lead to functional abnormalities at various levels, possibly explaining some of the clinical manifestations of COVID-19. The mechanism by which SARS-CoV-2 invades the CNS is not fully understood. Still, there are several theories, including retrograde transmission from the peripheral nervous system (PNS), hematologic spread, and transmission across the blood–brain barrier (BBB) [24]. 

**Figure 1:**
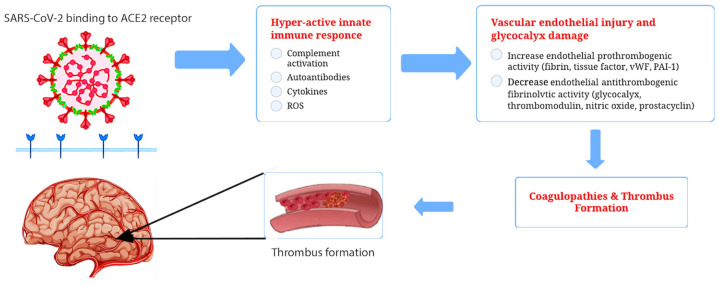
The SARS-CoV-2 virus may cause brain pathology through both direct and indirect mechanisms. There is evidence that ACE2, a functional receptor of SARS-CoV-2, can facilitate direct invasion into neurons and cerebrovascular endothelial cells, which can result in the apoptosis of neurons and surrounding cells. The SARS-CoV-2 infection also results in cytokine storms. Cytokine storms can damage an intact BBB and disrupt normal functioning in the brain. Additionally, COVID-19 has been associated with a prothrombotic state, which may result in the occlusion of cerebral vessels and brain damage.

The infection caused by SARS-CoV-2 specifically targets endothelial cells [25]. It has not yet been fully characterized how SARS-CoV-2 affects endothelial cells and its implications for apoptosis and function. However, viral bodies within the cells indicate that the virus is involved. The presence of viral components inside endothelial cells has been demonstrated [26] along with the accumulation in inflammatory cells, as well as evidence of both endothelial and inflammatory cell death. Viruses can use different mechanisms to harm the endothelium. Aside from causing apoptosis in endothelial cells, viruses also significantly increase cytokine levels, resulting in alterations to the cell junctions, increasing vascular permeability, and altering endothelial function [27]. As a result of COVID-19, endothelial cells are likely to detach rapidly, and cell regeneration may not occur as effectively as expected [28]. The endothelium becomes activated due to the cytokine storm, resulting in endothelial dysfunction, endothelial cell death, increased vascular permeability, and impaired endothelial barrier functionality, ultimately leading to cell detachment [27]. As a result, parts of the inner surface devoid of endothelial cells appear, and the detached endothelial cells enter the bloodstream. In recent studies [29], researchers have demonstrated that both adherent and detached endothelial cells become procoagulant. Coagulability is due to increased phosphatidylserine expression and an absence of anticoagulant components such as thrombomodulin and tissue factor (TF) pathway inhibitors. In the presence of TF, an extrinsic coagulation cascade is initiated, leading to disseminated, uncontrolled, and widespread intravascular coagulation [29]. Figure 1 illustrates SARS-CoV-2 receptor binding, the viral inflammatory response, vascular endothelial dysfunction, subsequent hypercoagulation, and thrombus formation. 

The cerebral vasculature can maintain a consistent blood flow despite alterations in cerebral perfusion pressure known as cerebral autoregulation [13]. Usually, cerebral blood flow is regulated by the diameter of arterioles, which influences cerebral blood flow (CBF) resistance. It remains unclear exactly how autoregulation is mediated at the molecular level [30]. Vasomotor responses are modulated by several processes including myogenic, neuro-genic, endothelial, and metabolic responses. The vascular endothelium is a crucial neurovascular unit (NVU), and it plays a fundamental role in regulating the blood–brain barrier and cerebrovascular reserve [31]. The endothelium also encompasses a substantial surface area responsible for regulating hemodynamic functions through the secretion of relaxing and contracting factors [25]. Vascular dysfunction manifests as an imbalance between the production of relaxing and contracting factors. Cerebrovascular reactivity (CVR) is a quantitative measure of NVU function. The CVR reflects NVU-mediated changes in cerebral blood flow in response to vasoactive stimuli.

This impairment of vascular function is likely a result of inflammation, the senescence of vascular cells, an increase in oxidative stress, reduced production or release of nitric oxide (NO) and other relaxing factors, as well as an increase in the production of vessel-contracting factors [32,33,34]. By losing endothelial cells on the luminal surface, normal reactivity mechanisms mediated by the endothelium, such as NO production, are disrupted [35]. Besides preventing abnormal contractions, NO inhibits platelet aggregation [36], suppresses the expression of adhesion molecules on endothelial cell surfaces, and therefore restricts white blood cell adhesion and penetration [28]. Decreasing NO production eliminates these protective effects of coagulation and inflammation [37]. Furthermore, disruption to the endothelial barrier allows aggregating platelets to approach vascular smooth muscle cells, triggering their contraction, which initiates the vascular phase of hemostasis. The breath-holding examination serves as a physiological test to assess ventilatory and metabolic responses during voluntary breath retention. This evaluation involves the deliberate cessation of respiration for a predetermined duration while closely monitoring various physiological reactions [38]. A primary objective of this test is to determine the body’s ability to regulate ventilation and metabolism. By assessing VMR through the breath-holding test (BHT), valuable insights are obtained into the intricate control mechanisms governing respiration and metabolism. The BHT for VMR assessment can assist in evaluating the autonomic nervous system’s response.

By analyzing the function of the endothelial cells, helpful information on the severity of the disease can be obtained since this disease rapidly affects the endothelial cells through various mechanisms. VMR serves as a critical marker of cerebral vascular function, particularly endothelial function, determining the ability of cerebral arteries to constrict or dilate in response to changes in carbon dioxide levels and resistance to blood flow within the brain. Assessing VMR can provide valuable insights into chronic endothelial dysfunction in populations at risk of experiencing long-term effects of COVID-19. Additionally, it can aid in closely monitoring COVID-19 patients to reduce the impact of the disease on high-risk patients.

## 3. Flow Assessment in the Brain following Infection with COVID-19

CVR is a term used to describe the ability of brain blood vessels to dilate or constrict when metabolic demands or the microenvironment change [13]. Throughout the brain, oxygen and nutrients are delivered to brain tissue by this mechanism, which ensures continuous cerebral perfusion. Cerebral circulation is sensitive to arterial pCO_2_ as it is a powerful vasomotor stimulus. Increased pCO_2_ and decreased pH raise cerebral blood flow (CBF), whereas increased pO2 has the opposite effect [12].

**Figure 2:**
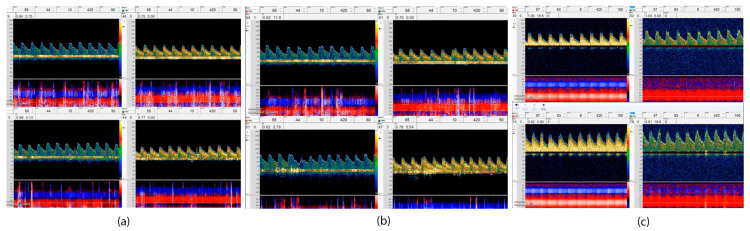
This figure illustrates using TCD to make a VMR assessment. This includes three VMR results (baseline study and follow-up) from a patient. A 52-year-old woman with no previous medical history was admitted to the hospital following a diagnosis of moderate COVID-19, and a brain imaging scan revealed that a scattered stroke had occurred in two hemispheres of her brain. All vessel imaging and stroke workup were unremarkable, (**a**) TCD showed abnormal VMR in the first week MFV in the right and left MCA (M1) was about 44 cm/s with no significant change after breath holding. (BHI—Right: 0.12 BHI—Left: −0.05); (**b**) VMR mildly improved in the third week, MFV in the right MCA increased from 41 cm/s to 44 cm/s and on the left MCA increased from 40 cm/s to 47 cm/s after breath holding (BHI—R: 0/12 and BHI—L: 0.5); (**c**) after full recovery in 12 weeks, the BH index recovered well with increasing in MFV on the right MCA from 55 cm/s to 74 cm/s and on the left side, from 45 cm/s to 76 cm/s after breath holding (0.38 on the right side and 0.45 on the left side).

CO_2_ reactivity, acetazolamide, and the BHT can be used in clinical practice to assess CVR [12]. An easy, noninvasive, reproducible, and reliable method can be obtained using carbon dioxide reactivity tests if the mixture of CO_2_ (3–7%) is inhaled for approximately 90 s [39]. The BHT induces hypercapnia via 30 s of apnea in order to calculate the breath-holding index (BHI) [40]. A breath BHI is calculated as a percentage increase in velocity from resting baseline values divided by the duration of breath holding (PSVmax—PSVrest/seconds) [41]. While BHT is noninvasive, easily performed, well-tolerated, and widely accepted, it has limited pCO_2_ changes (roughly 3–4 mm Hg), requires patient collaboration, and is less reproducible. According to Figure 2, three VMR results from a patient are illustrated through the use of TCD for BHI assessment.

As a final procedure, the Acetazolamide test involves the intravenous infusion of 500–2000 mg of carbonic anhydrase inhibitor, acetazolamide, which causes a transient marked amount of cerebral acidosis and vasodilation. Although this test is widely used due to its simplicity and absence of patient collaboration, it is less accurate and reproducible than the former, and it has undesirable side effects such as arterial hypertension, headaches, nausea, and perioral dysesthesia.

The response of cerebral vessels to vasoactive stimuli can be measured using various nuclear medicine and imaging techniques. PET is considered the gold standard for investigating CVR because it measures CBF directly. It is also possible to use near-infrared spectroscopy (NIRS), single photon emission computed tomography (SPECT), fMRI, CT with xenon enhancement, and transcranial Doppler sonography (TCD) [41,42,43]. TCD is a relatively reliable, inexpensive, widely available, and noninvasive method of measuring hemodynamic parameters in the main intracranial arteries, including peak systolic, diastolic, and mean velocity (MV) [44]. There are some limitations to TCD with transient vasodilator stimulations. Still, it is widely used in clinical practice to assess CVR and MV reduction indicating decreased global or regional CBF, pulsatility index (PI), and particularly, VMR [45]. CVR can be estimated by measuring changes in flow velocities in response to vasodilator stimuli in the main cerebral arteries as an indirect indication of changes in CBF. 

Recently, researchers have found that impaired VMR is associated with a higher probability of severe disease and poor outcomes in patients with COVID-19. A functional dynamic assessment of the vascular function is more sensitive than a structural or morphological assessment after acute exposure to the disease process or factors affecting the vascular conduits are present. The structural changes in vasculature occur more slowly than the functional changes [15]. Therefore, cerebrovascular function can be assessed to provide valuable information regarding the progression of the disease and to assist in selecting an appropriate treatment plan. Additionally, CVR has been observed to change over time in patients with different clinical neurologic manifestations, suggesting that it could serve as a biomarker for the progression of the disease [12].

We searched PubMed/MEDLINE, Google Scholar, and EMBASE from the database’s inception until 19 May 2023. Additionally, we manually searched the references of relevant articles. The following keywords were used in our search: VMR, vasoreactivity, cerebral hemodynamics, COVID-19, severe acute respiratory syndrome Coronavirus 2, CT perfusion, TCD, MRI, intracranial compliance, and perfusion imaging. After excluding non-English studies and case reports, seven articles were found and will be discussed in the present narrative review. A meta-analysis was not possible due to the heterogeneity in study design and reporting of outcomes; therefore, only studies were reviewed. To the best of our knowledge, seven articles [14,46,47,48,49,50,51] have evaluated VMR and PI in COVID-19 patients (Table 1). TCD was used for all of them except for Callen et al. [51], who used MRI to assess COVID-19 patients. Two studies evaluated the effects of infection up to four weeks after infection, two studies after six months, and the other studies assessed the impact between those two times. Apart from one study, all used healthy control subjects to compare hemodynamic findings.

A study by Marcic et al. [48] evaluated cerebral hemodynamics and BHI in 25 patients with mild COVID-19 experiencing non-specific neurological symptoms 40 days after receiving a negative result for SARS-CoV-2, compared to 25 healthy individuals recruited as controls. The study did not identify any significant risk factors for cerebrovascular disease, and the BHI was significantly lower in the infectious patients than in the control group, suggesting an impaired VMR. A similar finding has been reported in other studies that have demonstrated impaired VMR following infection with COVID-19 [14,46]. In another study, Abdo-Cuza et al. [47] compared cerebral hemodynamic reserve between two groups. The first group consisted of 25 recovered COVID-19 patients who suffered from varying degrees of disease severity and were free of neurological symptoms or diseases at the time of inclusion. Taking into account the severity of the disease and its impact on VMR, patients were further categorized into two groups: asymptomatic, mildly ill, and severely ill. The second group consisted of 26 individuals who had never been diagnosed with COVID-19 and had tested negative at enrollment. Their findings showed a lower CVR and BHI in those with COVID-19 compared with control participants (19.9% vs. 36.8%, and 0.7 vs. 1.2). It is noteworthy that these variables were similar among patients with asymptomatic (1.9%) or mild disease (19.8%) and those with severe and critical (0.7%) disease. An increase in endothelial dysfunction among patients who are asymptomatic or mildly infected with COVID-19 may impact a large percentage of the infected patients; however, their study population is relatively small, and further studies are necessary to test this hypothesis. In contrast, Nandadeva et al. [49] reported no significant differences in cerebral VMR between 16 young adults diagnosed with COVID-19 at least four weeks prior and 12 controls not diagnosed.

A global healthcare crisis and strain on healthcare resources have resulted from the COVID-19 pandemic. As the population of patients recovering from COVID-19 grows, it is paramount to understand the healthcare issues surrounding them [52,53]. COVID-19 is now recognized as a multi-organ disease with a broad spectrum of manifestations. Similar to post-acute viral syndromes described in survivors of other virulent coronavirus epidemics, there are increasing reports of persistent and prolonged effects after acute COVID-19. Many patients still experience physical, psychological, or cognitive symptoms after recovering from acute COVID-19 [54]. Long-COVID refers to the prolonged symptoms of SARS-CoV-2, which can involve a wide range of extrapulmonary organ dysfunction, including structural neurologic defects [55]. More patients require ‘long-COVID’ care, making it challenging for neurologists to keep up with the demands [56]. Most studies investigating post-acute COVID-19 clinical neurodegenerative disorders have been conducted on patients admitted during the acute phase of COVID-19, and very few studies have examined the long-term effects after the acute phase [56]. To integrate multispecialty care in the outpatient setting, COVID-19 clinics will need a comprehensive understanding of patient care needs beyond the acute phase. It is vital to construct post-acute COVID-19 care strategies and guide healthcare system capacity planning. To assess long-term consequences after COVID-19 infection, Marcic et al. [50] conducted a cross-sectional study of 49 individuals diagnosed with COVID-19 who were experiencing mild neurological symptoms 300 days following the onset of the disease, along with 50 controls of similar age and gender. The study found a statistically significant reduction in BHI among subjects who acquired COVID-19 infection compared to the control group, indicating chronic endothelial dysfunction. Assessing VMR through TCD may provide helpful information about chronic endothelial dysfunction in a population prone to long-COVID. It could be used to closely monitor COVID-19 patients with cerebrovascular diseases, as methods like MRI may not be accessible or repeatable.

## 4. The Impact of Infection Severity and Neurologic Symptoms on VMR

According to the early reports from Wuhan, China, 36% of infected patients suffer from neurological manifestations [57]. Since this information was released, there have been several multicenter cohort studies, comprehensive reviews, and meta-analyses investigating the neurological effects of COVID-19 [58,59]. Survivors of COVID-19 with neurologic involvement seem to be uniformly associated with poorer outcomes, including hospital admissions, mortality, and disability. Recent studies note that neurologic syndromes are not exclusively associated with the critically ill [60,61]. There are several significant cerebrovascular diseases, including acute cerebrovascular events (ischemic stroke, cerebral hemorrhage, subarachnoid hemorrhage), acute encephalopathy, encephalitis or meningitis, polyneuropathy, demyelinating spectrum of illness, and seizures, that can affect VMR and alter cerebral hemodynamics [62]. Our comprehensive literature review observed that among the studies we included that investigated patients infected with COVID-19, two of them recruited individuals who exhibited no neurological symptoms or pre-existing conditions at the time of their inclusion [47,49]. Additionally, two studies examined patients with non-specific neurological symptoms [48,50]. Moreover, one study focused on recruiting patients admitted to the intensive care unit [14], while another specifically targeted patients with acute ischemic stroke [51]. Notably, one of the studies did not mention cerebrovascular disease among its participants [46].

Callen et al. [51] was a unique study, as they included patients who experienced critical illness during their infection (acute ischemic stroke requiring hospitalization). The study examined CVR and vessel wall imaging in fifteen patients with COVID-19 and ten control participants. Of the infected, twelve were mildly ill (no acute neurovascular events, hospitalizations, or other critical illnesses) and three were critically ill. Furthermore, seven reported persistent neurologic symptoms for at least one month following the illness, including headache, memory impairment, insomnia, depression, disequilibrium, dysgeusia, and tinnitus. Prior infection was associated with a decrease in whole-brain CVR after excluding the three critically ill participants and adjusting for age and sex (*p* = 0.001). The CVR was lower in those with post-COVID neurologic conditions than those without, but the difference was not statistically significant (*p* = 0.22). This study’s attempt to quantify the impact of acute cerebrovascular disease and mild illness on VMR impairment is not feasible. This limitation arises from the small number of participants and the substantial variability in age and time elapsed since SARS-CoV-2 infection. Additionally, only a fraction of the participants (7 out of 15) reported experiencing neurological symptoms. Future studies with a more comprehensive design are necessary to understand the clinical implications of SARS-CoV-2-associated CVR impairment.

In a few studies investigating VMR disturbances in patients with COVID-19, most recruited patients without neurologic disease and excluded patients with significant risk factors for cerebrovascular disease [48,50]. Two studies included COVID-19 patients with non-specific neurological symptoms such as smell and taste dysfunction, vertigo, headache, dizziness, or fatigue, and excluded cerebrovascular disease. The authors did not recruit healthy control patients and did not examine the relationship between mild neurologic symptoms and VMR impairment. Those with persistent post-COVID neurologic conditions may have experienced heterogeneous symptoms, and SARS-CoV-2 infection has not been proven to cause these symptoms. More research is required to delineate the relationship between chronic CVR impairment and the chronic neuropsychologic sequelae of SARS-CoV-2 infection.

Only two studies examined the impact of infection severity on the VMR. The study conducted by Abdo-Cuza et al. [47] recruited two groups of patients. The first group consisted of twenty-five patients who recovered from COVID-19. The recovered patients were divided into two categories: those who suffered from mild or asymptomatic disease and those who suffered from severe or critical illness. The second group consisted of twenty-six patients who tested negative for COVID-19. All participants were asymptomatic at the time of enrollment into the study. The cerebral hemodynamic reserve and breath-holding index of patients who recovered from SARS-CoV-2 infection were decreased, regardless of the severity of the disease or the presence of neurological symptoms. This finding is likely consistent with the damage to cerebral microvasculature that occurs in various conditions, including COVID-19. Additional research is required to understand neurological signs and symptoms during the disease’s initial clinical presentation or recovery and how COVID-19’s clinical severity affects the VMR.

Another study recruited patients admitted to the ICU; however, they evaluated intracranial compliance (ICC) without assessing VMR [14]. The clinical outcomes of mechanically ventilated COVID-19 patients were examined in the context of cerebrovascular hemodynamics (CVH) and intracranial compliance (ICC). To assess CVH, the mean flow velocities in the middle cerebral arteries (mCBFV), pulsatility index (PI), and estimated cerebral perfusion pressure (eCPP) were used. To evaluate ICC, the ratio of P1/P2 of the non-invasive intracranial pressure curve (ICP) was utilized. Fifty critically ill COVID-19 patients were studied with TCD and noninvasive monitoring of ICC. CVH and ICC were assessed twice: once during the first three days following intubation and again within 72 h following extubation or tracheostomy without the administration of sedatives. The first evaluation was used only for patients who died while intubated. Most COVID-19 patients who were on a ventilator had abnormal brain hemodynamics. They used a combination of ICC and CVH measurements to investigate if there was a link between those values and patient outcomes. There was a significant difference between CVH/ICC scores for patients with a favorable outcome (*p* = 0.001) and those with an unfavorable outcome (UO). A UO was defined as failure to wean from respiratory support or death on day seven following weaning. This study had some limitations, including a small number of participants despite a large pool of COVID-19 patients, which may have led to selection bias. Additionally, neurological imaging was not conducted during the study period. Comprehensive data on ventilatory parameters such as PEEP, tidal volume, and plateau pressure were not accessible for all patients. There is no established validation for the CVH/ICC score that was devised, and it should be evaluated in large cohorts of critically ill patients.

## 5. MRI for Cerebral Blood Flow Assessment following COVID-19 Infection

Functional magnetic resonance imaging (fMRI) is a noninvasive neuroimaging technique that measures the hemodynamic response to neural activity in the brain. A critical component of the method is that changes in blood flow, mainly oxygenated blood, correlate with neural activity. In addition to providing valuable insights into neural mechanisms underlying vascular dysfunction in neurological diseases, fMRI has several advantages over traditional methods, such as TCD and positron emission tomography (PET). The technique is noninvasive, requires no ionizing radiation, and has a high spatial resolution, enabling detailed mapping of brain regions involved in vasomotor control. While this method has some advantages, it has certain limitations. One of these limitations is that it is a relatively slow technology, with a time resolution on the order of seconds, making it challenging to capture rapid changes in neural activity. A second limitation of fMRI scanners is that their magnetic field may be uncomfortable or even distressing for some individuals, particularly those who experience claustrophobia or anxiety. Furthermore, fMRI is an expensive technology requiring specialized equipment and expertise, which may limit its availability and accessibility to some researchers and patients. In a study by Callen and colleagues [51], the authors examined the CVR and vessel wall imaging of patients with prior COVID-19 (including seven individuals with post-COVID neurologic conditions) and ten control participants who had never had SARS-CoV-2 infection. The study utilized MRI that included arterial spin labeling perfusion imaging with acetazolamide stimulus. After adjusting for age and sex, a linear model was used to assess associations between CVR and prior infection. The difference in CVR between the two groups remained statistically significant even after excluding the three participants with previous illnesses who experienced an acute ischemic stroke and hospitalization after infection (*p* < 0.001). Although the difference was insignificant, the authors found that CVR was lower in individuals with post-COVID neurologic conditions than those without (16.9 vs. 21.0 mL/100 g/min; *p* = 0.22) (Table 1).

The authors concluded that SARS-CoV-2 infection is associated with chronic impairment of CVR, but the mechanistic basis of this chronic neurovascular endothelial dysfunction remains unknown.

## 6. Cerebral Blood Flow Evaluation following Other Virus Infections

Recent research has demonstrated that viruses such as Human Herpesvirus 8 and the Hantavirus predominantly affect endothelial cells and, therefore, affect cerebral hemodynamics [63,64,65]. A study by Grahame-Clarke et al. [16] demonstrated that people with human cytomegalovirus seropositivity have impaired endothelial function and impaired NO responses. Moreover, the effect of human immunodeficiency virus (HIV) on endothelial cells has been extensively studied [66]. VMR of the brain and hemodynamic changes following viral infections are not fully understood. However, some authors have suggested that these alterations may be due to direct viral invasion of the brain or indirect effects, such as inflammation and cytokines. The immune response to viral infection may also contribute to the alterations. To improve our understanding of the impact of viral infections on VMR and hemodynamic alterations, larger studies involving more diverse populations are necessary.

The assessment of the endothelium by VMR could aid in improving patient care during the acute or chronic phase of other viral infections. Further research is required to comprehend the mechanisms fully. Furthermore, efforts should be made to standardize the method used to measure cerebral blood flow velocity and cerebral autoregulation to allow for better comparison between studies.

## 7. Limitations and Strengths

Among the included studies, most had methodological or design shortcomings to some degree, such as inadequate randomization, insufficient blinding of participants and personnel, and small sample sizes. The authors did not include persistent symptoms and the severity of COVID-19 and underlying diseases. TCD used different stimulants to measure VMR: for example, the hemodynamic effect of breath holding is lower than that of carbon dioxide inhalation or the injection of acetazolamide. Additionally, TCD is user dependent and varying degrees of experience could have impacted the results. Despite these limitations, this review highlights several strengths. One such strength is that it generates recommendations for further research to confirm these findings, in order to help guide future policy choices.

## 8. Conclusions

Patients who had experienced COVID-19 infection showed cerebral hemodynamic impairment, regardless of initial infection severity and the absence of neurological symptoms. Most studies on this topic indicate that COVID-19 infection affects cerebral hemodynamics, which is reflected in VMR. Hemodynamic disturbances in the brain can result in a variety of health problems. A higher risk of severe disease and poor outcomes can be associated with impaired VMR. Using VMR, one can gain valuable insight into a patient’s disease progression and make more informed decisions regarding appropriate treatment options. Additional research is required to fully understand the role of VMR in COVID-19 and other viral infections. Despite this, the evidence indicates that this is an important area of investigation that may improve patient outcomes in the future. A new pandemic may develop, making it essential for healthcare providers and researchers to remain focused on developing new strategies to improve survival in patients, particularly those with cerebrovascular risk factors. The crucial role of VMR in this endeavor cannot be overstated.

## Figures and Tables

**Table 1:** Overview of the studies determining VMR in COVID-19 patients during the acute phase and following infection.

Author, Year	Participants (n)/Control (n)	COVID-19 Severity	Neurologic Complication	The Interval between Infection and Test	VMR Tool/Method/Implied Vessel	Outcome
Sonkaya et al. [46], 2020	COVID-19 patients (20)/healthy control (20)	Not intubated patients	NM	Immediately after hospitalization	TCD/BHT/MCA	The mean VMR values were significantly lower in the patient group compared to the control group.
A. Abdo-Cuza et al. [47], 2021	COVID-19 patients (25)/healthy control (26)	Asymptomatic to critical	Headache (3), loss of smell (2), and loss of taste (1). All were asymptomatic at the test time.	14 days	TCD/BHT/MCA	Patients recovered from SARS-CoV-2 infection showed decreased cerebral hemodynamic reserve and BHI regardless of the disease’s clinical severity or neurological symptoms.
Marcic et al. [48], 2021	COVID-19 patients (25)/healthy control (25)	Mild	All had post-infection non-specific neurological symptoms.	28–50 days	TCD/BHT/MCA	Patients had significantly lower BHI as compared to the control group.
Nandadeva et al. [49], 2021	COVID-19 patients (16)/healthy control (12)	Mild to moderate	Loss of smell and or taste (7), fatigue (1), severe muscle pain after exercise (1) at the test time.	28 days	TCD/hypercapnia/MCA	CVR was not different between groups.
Brasil et al. [14], 2021	UO COVID-19 patients (33)/FO COVID-19 patients (17)	Critically ill	NM	3 days	TCD/PI/MCA	Intracranial compliance impairment and CVH disturbances are often present in severe COVID-19 illness and could accurately predict an early poor outcome.
Marcic et al. [50], 2022	COVID-19 patients (49)/healthy control (50)	Mild	21 patients with mild non-specific neurological symptoms.	300 days	TCD/BHT/MCA	BHI in the patients group was lower than in the control group.
Callen et al. [51], 2022	COVID-19 patients (15)/healthy control (10)	Mild to severe	7 patients with post-COVID neurologic conditions.	238 days	MRI/acetazolamide	Patients had significantly lower whole-brain CVR than the control.

VMR, vasomotor reactivity; COVID-19, coronavirus disease 2019; NM, not mentioned; TCD, transcranial Doppler ultrasound; BHT, breath-holding test; MCA, middle cerebral artery; BHI, breath-holding index; CVR, cerebrovascular reactivity; UO, unfavorable outcome; FO, favorable outcome; PI, pulsatility index.

## Data Availability

Data is contained within the article.

## References

[1] Monari C., Gentile V., Camaioni C., Marino G., Coppola N., Vanvitelli COVID-19 group Vanvitelli COVID-19 group (2020). A Focus on the Nowadays Potential Antiviral Strategies in Early Phase of Coronavirus Disease 2019 (COVID-19): A Narrative Review. Life.

[2] Estakhr M., Tabrizi R., Ghotbi Z., Shahabi S., Habibzadeh A., Bashi A., Borhani-Haghighi A. (2022). Is Facial Nerve Palsy an Early Manifestation of COVID-19? A Literature Review. Am. J. Med. Sci..

[3] Novaes N., Sadik R., Sadik J.-C., Obadia M. (2022). Epidemiology and Management of Cerebral Venous Thrombosis during the COVID-19 Pandemic. Life.

[4] Ren A.L., Digby R.J., Needham E.J. (2021). Neurological Update: COVID-19. J. Neurol..

[5] Iadecola C., Anrather J., Kamel H. (2020). Effects of COVID-19 on the Nervous System. Cell.

[6] Orrù G., Conversano C., Malloggi E., Francesconi F., Ciacchini R., Gemignani A. (2020). Neurological Complications of COVID-19 and Possible Neuroinvasion Pathways: A Systematic Review. Int. J. Environ. Res. Public. Health.

[7] Helms J., Kremer S., Merdji H., Clere-Jehl R., Schenck M., Kummerlen C., Collange O., Boulay C., Fafi-Kremer S., Ohana M. (2020). Neurologic Features in Severe SARS-CoV-2 Infection. N. Engl. J. Med..

[8] Baig A.M., Khaleeq A., Ali U., Syeda H. (2020). Evidence of the COVID-19 Virus Targeting the CNS: Tissue Distribution, Host–Virus Interaction, and Proposed Neurotropic Mechanisms. ACS Chem. Neurosci..

[9] Pennisi M., Lanza G., Falzone L., Fisicaro F., Ferri R., Bella R. (2020). SARS-CoV-2 and the Nervous System: From Clinical Features to Molecular Mechanisms. Int. J. Mol. Sci..

[10] Shehata G.A., Lord K.C., Grudzinski M.C., Elsayed M., Abdelnaby R., Elshabrawy H.A. (2021). Neurological Complications of COVID-19: Underlying Mechanisms and Management. Int. J. Mol. Sci..

[11] Gladka M.M., Maack C. (2020). The Endothelium as Achilles’ Heel in COVID-19 Patients. Cardiovasc. Res..

[12] Staszewski J., Skrobowska E., Piusińska-Macoch R., Brodacki B., Stępień A. (2019). Cerebral and Extracerebral Vasoreactivity in Patients With Different Clinical Manifestations of Cerebral Small-Vessel Disease: Data From the Significance of Hemodynamic and Hemostatic Factors in the Course of Different Manifestations of Cerebral Small-Vessel Disease Study. J. Ultrasound Med..

[13] Claassen J.A.H.R., Thijssen D.H.J., Panerai R.B., Faraci F.M. (2021). Regulation of Cerebral Blood Flow in Humans: Physiology and Clinical Implications of Autoregulation. Physiol. Rev..

[14] Brasil S., Taccone F.S., Wayhs S.Y., Tomazini B.M., Annoni F., Fonseca S., Bassi E., Lucena B., Nogueira R.D.C., De-Lima-Oliveira M. (2021). Cerebral Hemodynamics and Intracranial Compliance Impairment in Critically Ill COVID-19 Patients: A Pilot Study. Brain Sci..

[15] Nakano H., Shiina K., Tomiyama H. (2021). Cardiovascular Outcomes in the Acute Phase of COVID-19. Int. J. Mol. Sci..

[16] Grahame-Clarke C., Chan N.N., Andrew D., Ridgway G.L., Betteridge D.J., Emery V., Colhoun H.M., Vallance P. (2003). Human Cytomegalovirus Seropositivity Is Associated With Impaired Vascular Function. Circulation.

[17] Hamming I., Timens W., Bulthuis M., Lely A., Navis G., Van Goor H. (2004). Tissue Distribution of ACE2 Protein, the Functional Receptor for SARS Coronavirus. A First Step in Understanding SARS Pathogenesis. J. Pathol..

[18] Zhang H., Penninger J.M., Li Y., Zhong N., Slutsky A.S. (2020). Angiotensin-Converting Enzyme 2 (ACE2) as a SARS-CoV-2 Receptor: Molecular Mechanisms and Potential Therapeutic Target. Intensive Care Med..

[19] Hassanzadeh K., Perez Pena H., Dragotto J., Buccarello L., Iorio F., Pieraccini S., Sancini G., Feligioni M. (2020). Considerations around the SARS-CoV-2 Spike Protein with Particular Attention to COVID-19 Brain Infection and Neurological Symptoms. ACS Chem. Neurosci..

[20] Arbour N., Day R., Newcombe J., Talbot P.J. (2000). Neuroinvasion by Human Respiratory Coronaviruses. J. Virol..

[21] Cheng Q., Yang Y., Gao J. (2020). Infectivity of Human Coronavirus in the Brain. EBioMedicine.

[22] Song E., Zhang C., Israelow B., Lu-Culligan A., Prado A.V., Skriabine S., Lu P., Weizman O.-E., Liu F., Dai Y. (2021). Neuroinvasion of SARS-CoV-2 in Human and Mouse Brain. J. Exp. Med..

[23] Hernández V.S., Zetter M.A., Guerra E.C., Hernández-Araiza I., Karuzin N., Hernández-Pérez O.R., Eiden L.E., Zhang L. (2021). ACE2 Expression in Rat Brain: Implications for COVID-19 Associated Neurological Manifestations. Exp. Neurol..

[24] Swain O., Romano S.K., Miryala R., Tsai J., Parikh V., Umanah G.K.E. (2021). SARS-CoV-2 Neuronal Invasion and Complications: Potential Mechanisms and Therapeutic Approaches. J. Neurosci..

[25] Xu S., Ilyas I., Weng J. (2023). Endothelial Dysfunction in COVID-19: An Overview of Evidence, Biomarkers, Mechanisms and Potential Therapies. Acta Pharmacol. Sin..

[26] Varga Z., Flammer A.J., Steiger P., Haberecker M., Andermatt R., Zinkernagel A.S., Mehra M.R., Schuepbach R.A., Ruschitzka F., Moch H. (2020). Endothelial Cell Infection and Endotheliitis in COVID-19. Lancet.

[27] Silva M.J.A., Ribeiro L.R., Gouveia M.I.M., Marcelino B.D.R., Santos C.S.D., Lima K.V.B., Lima L.N.G.C. (2023). Hyperinflammatory Response in COVID-19: A Systematic Review. Viruses.

[28] Six I., Guillaume N., Jacob V., Mentaverri R., Kamel S., Boullier A., Slama M. (2022). The Endothelium and COVID-19: An Increasingly Clear Link Brief Title: Endotheliopathy in COVID-19. Int. J. Mol. Sci..

[29] Bombeli T., Karsan A., Tait J.F., Harlan J.M. (1997). Apoptotic Vascular Endothelial Cells Become Procoagulant. Blood.

[30] Estakhr M., Ghotbi Z., Borhani-Haghighi A., Tarpley J.W., Bavarsad Shahripour R. (2022). Symptomatic Cerebral Vasospasm After Transsphenoidal Adenoma Resection of the Pituitary. J. Neurol. Res..

[31] Muoio V., Persson P.B., Sendeski M.M. (2014). The Neurovascular Unit - Concept Review. Acta Physiol..

[32] Hink U., Li H., Mollnau H., Oelze M., Matheis E., Hartmann M., Skatchkov M., Thaiss F., Stahl R.A.K., Warnholtz A. (2001). Mechanisms Underlying Endothelial Dysfunction in Diabetes Mellitus. Circ. Res..

[33] Maiuolo J., Mollace R., Gliozzi M., Musolino V., Carresi C., Paone S., Scicchitano M., Macrì R., Nucera S., Bosco F. (2020). The Contribution of Endothelial Dysfunction in Systemic Injury Subsequent to SARS-Cov-2 Infection. Int. J. Mol. Sci..

[34] Chang R., Mamun A., Dominic A., Le N.-T. (2021). SARS-CoV-2 Mediated Endothelial Dysfunction: The Potential Role of Chronic Oxidative Stress. Front. Physiol..

[35] Basta G. (2021). Direct or Indirect Endothelial Damage? An Unresolved Question. EBioMedicine.

[36] Kandhaya-Pillai R., Yang X., Tchkonia T., Martin G.M., Kirkland J.L., Oshima J. (2022). TNF -α/ IFN -γ Synergy Amplifies Senescence-associated Inflammation and SARS-CoV -2 Receptor Expression via Hyper-activated JAK / STAT1. Aging Cell.

[37] Münzel T., Daiber A., Ullrich V., Mülsch A. (2005). Vascular Consequences of Endothelial Nitric Oxide Synthase Uncoupling for the Activity and Expression of the Soluble Guanylyl Cyclase and the CGMP-Dependent Protein Kinase. Arterioscler. Thromb. Vasc. Biol..

[38] Cohen A.D., Wang Y. (2019). Improving the Assessment of Breath-Holding Induced Cerebral Vascular Reactivity Using a Multiband Multi-Echo ASL/BOLD Sequence. Sci. Rep..

[39] Ringelstein E.B., Van Eyck S., Mertens I. (1992). Evaluation of Cerebral Vasomotor Reactivity by Various Vasodilating Stimuli: Comparison of CO _2_ to Acetazolamide. J. Cereb. Blood Flow Metab..

[40] Kidwell C.S., El-Saden S., Livshits Z., Martin N.A., Glenn T.C., Saver J.L. (2001). Transcranial Doppler Pulsatility Indices as a Measure of Diffuse Small-Vessel Disease. J. Neuroimaging.

[41] Herrera Campos C.R., Beltramini G.C., Avelar W.M., Lima F.O., Li L.M. (2016). Cerebral Vasomotor Reactivity Assessment Using Transcranial Doppler and MRI with Apnea Test. Braz. J. Med. Biol. Res..

[42] Herzig R., Hluštík P., Školoudík D., Šaňák D., Vlachová I., Heřman M., Kaňovský P. (2008). Assessment of the Cerebral Vasomotor Reactivity in Internal Carotid Artery Occlusion Using a Transcranial Doppler Sonography and Functional MRI. J. Neuroimaging.

[43] Rijbroek A., Boellaard R., Vriens E.M., Lammertsma A.A., Rauwerda J.A. (2009). Comparison of Transcranial Doppler Ultrasonography and Positron Emission Tomography Using a Three-Dimensional Template of the Middle Cerebral Artery. Neurol. Res..

[44] Shahripour R.B., Azarpazhooh M.R., Akhuanzada H., Labin E., Borhani-Haghighi A., Agrawal K., Meyer D., Meyer B., Hemmen T. (2021). Transcranial Doppler to Evaluate Postreperfusion Therapy Following Acute Ischemic Stroke: A Literature Review. J. Neuroimaging.

[45] Ali M.F.A. (2021). Transcranial Doppler Ultrasonography (Uses, Limitations, and Potentials): A Review Article. Egypt. J. Neurosurg..

[46] Sonkaya A.R., Öztürk B., Karadaş Ö. (2021). Cerebral Hemodynamic Alterations in Patients with Covid-19. Turk. J. Med. Sci..

[47] Abdo-Cuza A.A., Hall-Smith C., Suárez-López J., Castellanos-Gutiérrez R., Blanco-González M.Á., Machado-Martínez R., Pi-Ávila J., Gómez-Peire F., Espinosa-Nodarse N., López-González J.C. (2021). Cerebral Hemodynamic Reserve Abnormalities Detected Via Transcranial Doppler Ultrasound in Recovered COVID-19 Patients. MEDICC Rev..

[48] Marcic M., Marcic L., Marcic B., Capkun V., Vukojevic K. (2021). Cerebral Vasoreactivity Evaluated by Transcranial Color Doppler and Breath-Holding Test in Patients after SARS-CoV-2 Infection. J. Pers. Med..

[49] Nandadeva D., Young B.E., Stephens B.Y., Grotle A.-K., Skow R.J., Middleton A.J., Haseltine F.P., Fadel P.J. (2021). Blunted Peripheral but Not Cerebral Vasodilator Function in Young Otherwise Healthy Adults with Persistent Symptoms Following COVID-19. Am. J. Physiol.-Heart Circ. Physiol..

[50] Marcic M., Marcic L., Lovric Kojundzic S., Marinovic Guic M., Marcic B., Caljkusic K. (2022). Chronic Endothelial Dysfunction after COVID-19 Infection Shown by Transcranial Color-Coded Doppler: A Cross-Sectional Study. Biomedicines.

[51] Callen A.L., Tanabe J., Thaker A.A., Pollard R., Sauer B., Jones W., Pattee J., Steach B., Timpone V.M. (2023). Evaluation of Cerebrovascular Reactivity and Vessel Wall Imaging in Patients With Prior COVID-19: A Prospective Case-Control MRI Study. Am. J. Roentgenol..

[52] Mathioudakis A.G., Fally M., Hashad R., Kouta A., Hadi A.S., Knight S.B., Bakerly N.D., Singh D., Williamson P.R., Felton T. (2020). Outcomes Evaluated in Controlled Clinical Trials on the Management of COVID-19: A Methodological Systematic Review. Life.

[53] Boaventura P., Macedo S., Ribeiro F., Jaconiano S., Soares P. (2022). Post-COVID-19 Condition: Where Are We Now?. Life.

[54] Schambeck S.E., Mateyka L.M., Burrell T., Graf N., Brill I., Stark T., Protzer U., Busch D.H., Gerhard M., Riehl H. (2022). Two-Year Follow-Up on Chemosensory Dysfunction and Adaptive Immune Response after Infection with SARS-CoV-2 in a Cohort of 44 Healthcare Workers. Life.

[55] Al-Aly Z., Xie Y., Bowe B. (2021). High-Dimensional Characterization of Post-Acute Sequelae of COVID-19. Nature.

[56] Xu E., Xie Y., Al-Aly Z. (2022). Long-Term Neurologic Outcomes of COVID-19. Nat. Med..

[57] Mao L., Jin H., Wang M., Hu Y., Chen S., He Q., Chang J., Hong C., Zhou Y., Wang D. (2020). Neurologic Manifestations of Hospitalized Patients With Coronavirus Disease 2019 in Wuhan, China. JAMA Neurol..

[58] Ray S.T.J., Abdel-Mannan O., Sa M., Fuller C., Wood G.K., Pysden K., Yoong M., McCullagh H., Scott D., McMahon M. (2021). Neurological Manifestations of SARS-CoV-2 Infection in Hospitalised Children and Adolescents in the UK: A Prospective National Cohort Study. Lancet Child Adolesc. Health.

[59] Kremer S., Lersy F., Anheim M., Merdji H., Schenck M., Oesterlé H., Bolognini F., Messie J., Khalil A., Gaudemer A. (2020). Neurologic and Neuroimaging Findings in Patients with COVID-19: A Retrospective Multicenter Study. Neurology.

[60] Voruz P., Allali G., Benzakour L., Nuber-Champier A., Thomasson M., Jacot De Alcântara I., Pierce J., Lalive P.H., Lövblad K.-O., Braillard O. (2022). Long COVID Neuropsychological Deficits after Severe, Moderate, or Mild Infection. Clin. Transl. Neurosci..

[61] Estakhr M., Ghotbi Z., Rostamihosseinkhani M., Hooshmandi E., Janipour M., Ostovan V.R., Fadakar N., Bazrafshan H., Bahrami Z., Rahimi-Jaberi A. (2023). The Hospitalization Rate and Clinical Characteristics of Mucormycosis Prior and during COVID-19 Pandemic: A Single-Center Study. J. Infect. Dev. Ctries..

[62] Matschke J., Krasemann S., Altmeppen H.C., Shafiq M., Glatzel M. (2022). NeuroCOVID: Insights into Neuroinvasion and Pathophysiology. Clin. Transl. Neurosci..

[63] Dupin N., Fisher C., Kellam P., Ariad S., Tulliez M., Franck N., Van Marck E., Salmon D., Gorin I., Escande J.-P. (1999). Distribution of Human Herpesvirus-8 Latently Infected Cells in Kaposi’s Sarcoma, Multicentric Castleman’s Disease, and Primary Effusion Lymphoma. Proc. Natl. Acad. Sci. USA.

[64] Mackow E., Gavrilovskaya I. (2009). Hantavirus Regulation of Endothelial Cell Functions. Thromb. Haemost..

[65] Steinberg B.E., Goldenberg N.M., Lee W.L. (2012). Do Viral Infections Mimic Bacterial Sepsis? The Role of Microvascular Permeability: A Review of Mechanisms and Methods. Antiviral Res..

[66] Singer E.J., Valdes-Sueiras M., Commins D.L., Yong W., Carlson M. (2013). HIV Stroke Risk: Evidence and Implications. Ther. Adv. Chronic Dis..

